# Application of response surface methodology for optimization of the test condition of oxygen evolution reaction over La_0.8_Ba_0.2_CoO_3_ perovskite-active carbon composite

**DOI:** 10.1038/s41598-023-49836-1

**Published:** 2023-12-18

**Authors:** Elham Mahmoudi, Elnaz Asghari, Nagihan Delibaş, Aligholi Niaei

**Affiliations:** 1 Department of Chemical and Petroleum Engineering, University of Tabriz, Tabriz, 5166616471 Iran; 2 Department of Physical Chemistry, Faculty of Chemistry, University of Tabriz, Tabriz, Iran; 3 Department of Physics, Faculty of Art and Science, Sakarya University, Sakarya, Turkey

**Keywords:** Chemistry, Engineering, Materials science

## Abstract

The Experimental Design was applied to optimize the electrocatalytic activity of La_0.8_Ba_0.2_CoO_3_ perovskite oxide/Active Carbon composite material in the alkaline solution for the Oxygen Evolution Reaction. After the preparation of La_0.8_Ba_0.2_CoO_3_, and structural characterizations, the experimental design was utilized to determine the optimal amount of the composite material and testing conditions. The overpotential was defined as the response variable, and the mass ratio of perovskite/active carbon, Potassium hydroxide (KOH) concentration, and Poly(vinylidene fluoride) (PVDF) amount were considered effective parameters. The significance of model terms is demonstrated by *P*-values less than 0.0500. The proposed prediction model determined the optimal amounts of 0.665 mg of PVDF, a KOH concentration of 0.609 M, and A perovskite/Active Carbon mass ratio of 2.81 with 308.22 mV overpotential (2.27% greater than the actual overpotential). The stability test of the optimized electrode material over 24 h suggests that it could be a good candidate electrocatalyst for OER with reusability potential.

## Introduction

Due to the exponential growth of population, increasing energy demand, and the limitation of non-renewable energy sources, research into finding renewable and environmentally-friendly energy alternatives, such as hydrogen, has become widespread^1–4^. Clean energy production processes, including electrochemical water splitting^5^, and Reversible Fuel Cells (RFCs), which employ electrochemical processes for hydrogen production ^6^, as well as energy storage devices like rechargeable metal-air batteries, known for their environmentally friendly, cost-effective, and high energy density^7^ , are crucial for achieving a sustainable energy perspective. Nevertheless, further developing these technologies requires enhancements, because there are some electrochemical drawbacks^8^ that need to be addressed, particularly the slow kinetics of the Oxygen Evolution Reaction (OER)^9,10^. OER holds significant importance in energy production and storage systems. At standard temperature and pressure, the thermodynamic potential for water splitting is 1.23 V^11^. However, OER is a four- electron charge transfer process that requires a considerable additional potential (overpotential) compared to the thermodynamic potential of the reaction. This overpotential is typically attributed to inherent reaction activation barriers and resistance, and its kinetics strongly depend on the electrocatalysts used to facilitate the reaction in both acidic and alkaline solutions^12^. Consequently, it is highly desirable to develop efficient and cost-effective electrocatalysts to enhance the OER process.

In the field of electrocatalysts for the OER, carbon materials^13–15^ have gained widespread usage owing to their high electronic conductivity, affordability, and structural versatility^16–19^. Nevertheless, their application is encumbered by challenges related to corrosion susceptibility^16,20^, the propensity to reduce the active surface area, and the formation of CO_x_ species at elevated potentials, factors that significantly curtail their practical suitability, particularly in industrial contexts^11,21^.

Currently, high-surface-area and nano-sized Iridium Oxide (IrO_2_), achieving a maximum current density of 1300 mA/cm^2^ at 1.8 V at 80 °C^22^. Meanwhile, Ruthenium Oxide (RuO_2_) demonstrates a maximum current density of 180 mA/cm^2^ at 1.8 V in acidic media. This is confirmed by chronoamperometric measurements that show RuO_2_’s stability for up to 20 h^23^. These materials are acknowledged as benchmark OER electrocatalysts^24,25^. Furthermore, the IrO_x_:RuO_y_ binary composite has emerged as an effective OER electrocatalyst, reaching a maximum current density of 146 mA/cm^2^ at 1.44 V versus NHE with a 24 h stability profile^26^. When placed on the volcano plot, these metal oxides exhibit a spread of data points, reflecting the interplay between OER activity and overpotential. It is evident that some metal oxides excel in OER activity, providing a high Turnover Frequency (TOF) or current density (J) at modest overpotentials. Others may display a lower overpotential but compromise on activity^27^. However, their non-selective nature for Chlorine-evolution reaction (CER) and OER in the water splitting process, limited resources, and their high prices have motivated researchers to seek more available, non-toxic, eco-friendly, selective, and low-cost electrocatalysts^28,29^. Transition metal oxides, conversely, are emerging as a highly promising class of OER electrocatalysts, driven by their cost-effectiveness, adjustable electrical conductivity, and remarkable catalytic activity for OER processes ^30,31^. Among them, Perovskite oxide with ABO_3_ structure ^32^, serve as attractive candidates for electrochemical applications^33^, especially OER in alkaline solutions ^34–44^; it is due to the unique physicochemical properties, presence of non-precious materials in the structure, being highly flexible in replacing elements in A and B sites ^45–47^, great intrinsic catalytic activity^48–50^ and chemical stability ^51^. The inclusion of perovskite oxides on the same volcano plot reveals intriguing insights into their OER activity. Notably, several perovskite oxides are positioned on the plot with distinct characteristics. A distinctive trend emerges, with certain perovskite oxides demonstrating remarkable OER activity at relatively low overpotentials. This suggests that these materials may possess inherent catalytic properties that hold great potential for reducing the energy barrier associated with the OER. Such behavior challenges the conventional trade-off between overpotential and activity and points toward new avenues for electrocatalyst development^52–54^. Research has indicated that Ba-based perovskites^55^ exhibit greater activity as OER electrocatalysts when compared to La_0.2_Sr_0.8_CoO_3_^56^, La_1−x_Ca_x_FeO_3_^57^, La_1−x_Sr_x_CoO_3_^58^, La_1−x_Ce_x_NiO_3_^59^, LaNi_0.8_Fe_0.2_O_3_^60^ in alkaline media. However, due to the high calcination temperature of perovskites, these oxides suffer low electrical conductivity, surface area and inadequate physical stability at room temperature, which restricts their utilization as commercial electrocatalysts^61,62^. To address this issue, Shao-Horn and co-workers have conducted a comprehensive investigation into the trends related to the Oxygen Reduction Reaction (ORR) and OER activities of perovskite oxides. Their work has introduced fundamental design principles for perovskite-based electrocatalysts. Particularly, the significance of maximizing the surface area of perovskite catalysts and its direct influence on their activity, especially concerning OER, was emphasized by their research group ^63,64^. Furthermore, Wang has emphasized that a larger surface area implies an increased number of active sites ^65^. In addition to enhancing the perovskite structure and augmenting the surface area, addressing the mentioned limitations involves the incorporation of conductive carbon as a current-conducting component and a polymeric binder ^66^. Carbon-based materials, known for their role as OER electrocatalysts, can serve as a conduit between the active surface of perovskite oxides without actively participating in the OER ^67,68^. A substantial amount of effort has been dedicated to the production of composites consisting of perovskite and carbon materials, with physical mixing being a prominent approach. For instance, the electrochemical OER performance of physically mixed composites, such as La_1−x_Ca_x_FeO_3_ and vulcan carbon (XC-72R)^57^, Ln_0.5_Ba_0.5_CoO_3_ (Ln: Pr, Sm, Gd and Ho) and acetylene black carbon^69^, LaFeO_3_ and Super P Li ^70^, LaCo_0.2_Fe_0.8_O_3_ and carbon black ^71^, as well as NdBaMn_2_O_5.5_ and vulcan XC-72 carbon^71^ composites has been investigated by various research groups. The synthesis of carbon nanotubes (CNT_s_) through Chemical Vapor Deposition (CVD) offers a promising avenue for creating composite materials with perovskite oxides for use as electrocatalysts in the OER^62,72^. Notably, Elumeeva and colleagues have demonstrated the potential of this technique by growing nitrogen-doped CNT_s_ (NCNT_s_) on the surface of an A-site-deficient La_0.58_Sr_0.4_Fe_0.2_Co_0.8_O_3_ perovskite oxide. In this arrangement, the B-site metal ions in the perovskite oxide serve as effective catalysts for both the OER/ORR^73^. In another approach, a composite of LaNiO_3_ and CNT_s_ nanohybrids has been prepared for OER electrocatalysis. To achieve this, perovskite oxides were employed as support materials, and a ferrocene-dissolved ethylenediamine solution was introduced as a precursor for the CNT_s_. This composite was fabricated using an injection CVD method, paving the way for improved OER electrocatalytic performance^74,75^. An alternative approach to enhancing the electrocatalytic activity for the OER is the synthesis of various materials using both hydrothermal and electrospinning methods. These materials include the LaNiO_3_-stabilized nitrogen and sulfur-codoped graphene (LaNiO_3_/N,S-Gr hybrid)^76^, LaNiO_3_ nanorod/reduced graphene oxide (RGO)^77^, and La(Co_0.55_Mn_0.45_)_0.99_O_3_ nanorod/nitrogen-doped reduced graphene oxide (NrGO)^78^, all produced through hydrothermal processes. Additionally, the synthesis of LaTi_0.65_Fe_0.35_O_3_ nanorods within nitrogen-doped carbon nanorods using electrospinning has been employed^79^. These methods have proven effective in enhancing the efficiency and performance of electrocatalysts in OER applications. Among these methods, the physical mixing approach is considered the most extensively researched method for preparing composite materials, mainly due to its potential for large-scale production. Historically, the incorporation of carbon through physical mixing was commonly thought to primarily function as a conductive support. Its main purpose was to increase the apparent electrical conductivity of the electrode, ultimately enhancing the overall utilization of perovskite materials^80^. On the other hand, adding Polyvinylidene fluoride (PVDF), with good physical stability and chemical resistance, has been recommended to prepare electrode materials^81,82^. Meanwhile, the relationship between the percentage of conductivity and OER activity makes it essential to find an optimal composition of electrode material^83^. To the best of our knowledge, notwithstanding the importance of the composition ratio on the electrochemical performance, no published articles used optimization methods to find the optimal composition and test condition at room temperature. The experimental design method is one well-known approach to study the effective parameters in different processes in which the effect of parameters can be investigated simultaneously^84,85^. Among these methodologies, Response Surface Methodology (RSM) plays a vital role in designing and optimizing the catalysts, which leads to improving the particular processes that contain different input variables. It is a set of mathematical and statistical techniques to explain a data set's behaviour to make statistical Predictions^86,87^. In our prior investigation, we synthesized a series of La_1−x_Ba_x_CoO_3_ perovskite oxides using the sol–gel method, with surface areas ranging from 4 to 14 m^2^/g. However, our electrochemical analysis revealed that when x = 0.2, producing the highest surface area, it exhibited unsatisfactory performance in the OER compared to BaCoO_3_ perovskite materials^55^. In this study, our objective is to explore the influence of various parameters on the electrocatalyst's performance. These parameters include the perovskite-to-carbon material ratio, the amount of PVDF in the composite, and the concentration of the KOH (Potassium hydroxide) solution. Therefore, our work fills an important gap in the existing literature.

This study aims to find an optimal amount of composite material and electrolyte concentration as effective parameters in OER utilizing RSM and Central Composite Design (CCD). The experimental design (DOE) method investigated three critical and effective parameters in the catalyst performance; the perovskite-to-carbon material ratio, electrolyte concentration, and binder quantity at room temperature. The response variable examined in this study was the overpotential required to achieve a current of 10 mA/cm^2^ in the OER. Following the synthesis of the perovskite as an active electrocatalyst, various structural analyses, including XRD, SEM, EDS, XPS, TEM, FT-IR, and N_2_ Adsorption–Desorption analyses, were done to study the structure of the catalysts. Subsequently, all 20 experiments proposed by Experimental Design were performed to determine the optimum condition for fabricating the electrode material to reach minimum overpotential.

## Materials and methods

### Preparation of the electrode material

Herein La_0.8_Ba_0.2_CoO_3_ perovskite oxide was employed as active material. For preparation of La_0.8_Ba_0.2_CoO_3_, La (NO_3_)_3_·6H_2_O, Ba(NO_3_)_2_·3H_2_O, and Glycine, from Samchun Pure Chemical Company, Co(NO_3_)_2_·4H_2_O, from Merck Company and de-ionized water were used as starting material and the sol–gel technique was employed to synthesis the perovskite oxide^88^.

Activated carbon (AC) from Sigma-Aldrich and PVDF from Tetiran Company were utilized to prepare the composite. The electrode materials were prepared by varying the mass ratio of La_0.8_Ba_0.2_CoO_3_/AC and the quantity of binder as proposed by RSM. To achieve a homogenous suspension, the solid materials were dispersed in N-Methyl-2-Pyrrolidone (NMP) (Merk Company), and the ink sonicated for 1 h.

### Structural characterization of electrocatalyst

To identify the crystalline phase of the perovskite and AC, X-ray diffraction analysis was performed in the Tongda TD-3700 (China) using Cu Kα radiation (λ = 1.5406 Å) between 20° and 75°. The morphological studies of samples were carried out using a scanning electron microscope (SEM) by Tescan instrument and Transmission Electron Microscopy (TEM) using CM120 (Netherlands). The quantitative evaluation of perovskite's composition was performed using Energy dispersive spectroscopy (EDS). The BET surface area and the textural properties of the perovskite were determined by N_2_ adsorption/desorption isotherms obtained using the TriStar II 3020 system. To characterize the functional groups of the surface, FT-IR spectra of the samples were obtained using a Bruker Model Tensor 27 spectrometer. The X-ray photoelectron spectroscopy (XPS) measurements were performed using a PHI 5000 VersaProbe, equipped with an Al Kα monochromatic source and a magnetic lens system. The binding energies of the acquired spectra were referenced to the C1s line at 284.9 eV.

### Electrochemical measurements

The electrochemical experiments were conducted under ambient conditions using a three-electrode setup. A homemade glassy carbon electrode (GCE) with 0.215 cm^2^ surface area, Pt wire, and Ag/AgCl severed as the working, counter, and reference electrodes. To ensure the removal of any impurities, the GCE was polished with Alumina powder prior to the fabrication of the working electrode. The electrode material was prepared according to the procedure described in the previous section employing various mass ratios of La_0.8_Ba_0.2_CoO_3_/AC ranging from 1 to 4 and varying amounts of PVDF ranging from 0.25 to 1 mg. These parameters ranges were selected based on sieved tests conducted before the optimization process. Subsequently, 6 µL of the ink was dropped over the electrode, and after drying for 24 h in the air, it was used as the working electrode. Polarization curves were obtained using the Linear Sweep Voltammetry (LSV) technique in KOH solution with concentrations ranging from 0.1 to 1 M and a scan rate of 10 mV/s. The curves were corrected based on Reversible Hydrogen Electrode (RHE) (Eq. 1), and the overpotential (η) was determined using Eq. (2)^89^.

1 $${{\text{E}}}_{{\text{RHE}}}={{\text{E}}}_{{\text{SCE}}}+{{\text{E}}}_{0{\text{SCE}}}+0.059*{\text{pH}}$$

2 $$\upeta ={{\text{E}}}_{{\text{experimental}}}-{{\text{E}}}_{{\text{thermodynamic}}}$$

### Experimental design

In this study, RSM based on the CCD was employed using Design Expert 12.0.3.0 software to model and optimize the experiments. The primary objective of this optimization approach was to achieve the optimal level of system variables, thereby maximizing performance^90^. Three factors include the mass ratio La_0.8_Ba_0.2_CoO_3_ /AC (A), the electrolyte material concentration (B), and the amount of PVDF (C). These factors were defined in five levels, as presented in Table 2, where 1, 0, and + 1 corresponded to low, central, and high levels, respectively. Furthermore, two levels beyond the cubic domain (Alpha) were defined to include six replicates at the central point, six at axial points, and eight at factorial points to predicate the response function. The response function assumed in this study was the required overpotential to reach 10 mA/cm^2^ current density during OER, denoted as (D). Design Expert software generated 20 experimental trials by varying of aforementioned variables. The validity of the model was evaluated using F-value and P-value. To establish a relationship between independent and response variables, a second-order model was employed (Eq. 3), where D_p_ represents the predicted response (overpotential (mV)), x_i_ and x_j_ denoted independent variables, x_i_x_j_ represents the interaction of xi and x_j_, β_i_, β_ii_, and β_ij_ show linear, quadratic, and interaction coefficients, respectively. β_0_ corresponds to the intercept value, k is the number of factors considered, and ε represents modeling error^91,92^.

3 $${{\text{D}}}_{{\text{P}}}=\sum_{{\text{i}}=1}^{{\text{k}}}{\upbeta }_{{\text{ii}}}{{\text{x}}}_{{\text{i}}}^{2}+\sum \sum_{{\text{i}}<{\text{j}}}{\upbeta }_{{\text{ij}}}{{\text{x}}}_{{\text{i}}}{{\text{x}}}_{{\text{j}}}+\sum_{{\text{i}}=1}^{{\text{k}}}{\upbeta }_{{\text{i}}}{{\text{x}}}_{{\text{i}}}+{\upbeta }_{0}+\upvarepsilon$$

## Results and discussion

### Structural characterization

**Figure 1:**
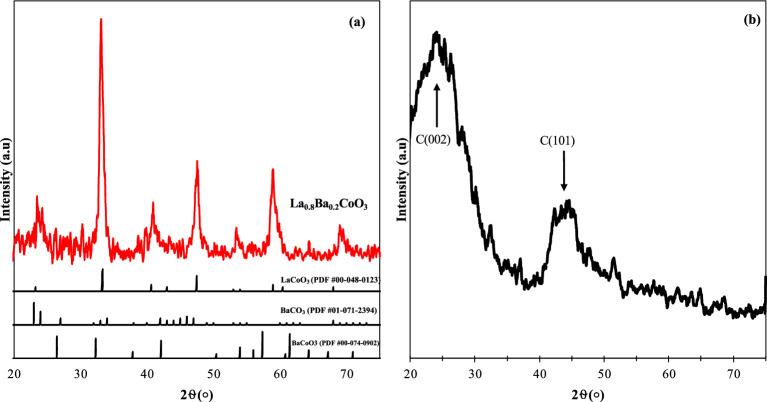
XRD Patterns of synthesized perovskite and references (**a**), and AC (**b**).

Figure 1 illustrates the XRD patterns of La_0.8_Ba_0.2_CoO_3_ and AC. Upon comparing the XRD pattern of La_0.8_Ba_0.2_CoO_3_ with the ICDD PDF database entries for LaCoO_3_, La_0.5_Ba_0.5_CoO_3_, and BaCoO_3_, it is evident that La_0.8_Ba_0.2_CoO_3_ exhibits a combination of these two perovskite phases^88,93^. Additionally, BaCO_3_ was formed within the perovskite structure as a side phase. It is noteworthy that the decomposition temperature of BaCO_3_ is above 800°C, whereas the synthesized perovskite was calcined at a lower temperature (650°C), resulting in the formation of BaCO_3_, which can be attributed to incomplete decomposition^94^.

Regarding the XRD pattern of AC, the prominent peaks observed at 2θ between 10 and 30° (the broad C (002) diffraction peak) and 40–50° (the weak and broad C (101) diffraction peak) can be attributed to the presence of graphite in the AC structure. Furthermore, noisy and disorderly diffraction signals indicate AC’s amorphous nature^95^.

**Figure 2:**
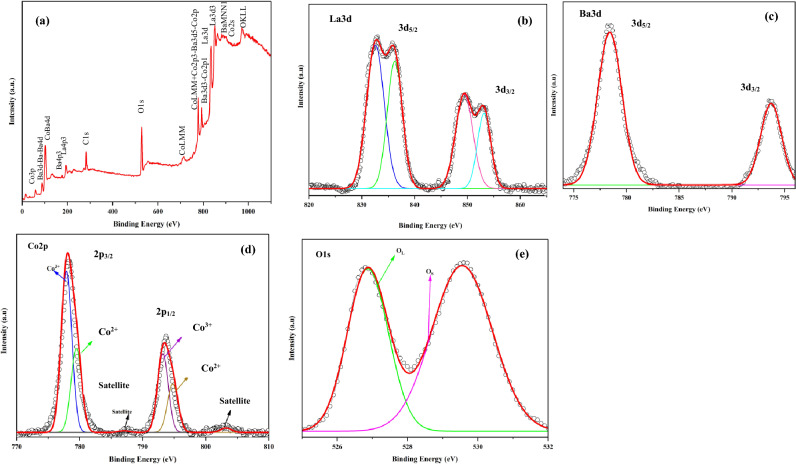
High-resolution of XPS spectra of La_0.8_Ba_0.2_CoO_3_ perovskites. (**a**) survey spectra, (**b**) La3d, (**c**) Ba3d, (**d**) Co2p, (**e**) O1s.

The surface properties of the La_0.8_Ba_0.2_CoO_3_ catalyst were thoroughly examined through XPS analysis. The composition of the La_0.8_Ba_0.2_CoO_3_ sample was unequivocally confirmed using XPS, as demonstrated in Fig. 2a, which presents the survey pattern of the sample. The XPS survey scan clearly identifies the presence of La, Co, Ba, and O elements on the surface of the material.

High-resolution XPS spectra of La3d, Ba3d, Co2p, and O1s were obtained, shedding light on the formal oxidation states of these elements. Specifically, the high-resolution La3d spectrum (Fig. 2b) exhibited two strong peaks at 832.57 and 849.36 eV, corresponding to the spin–orbit splitting of 3d_5/2_ and 3d_3/2_ orbitals of La^3+^ ions in oxide form. Furthermore, each of these peaks exhibited additional substructure, attributed to energy loss phenomena or “shake-up” satellites. The presence of two pairs of peaks in the La 3d_5/2_ spectrum (832.57, and 836.26 eV) indicates the coexistence of La^3+^ species in distinct surface environments^96,97^.

Moving on to the Co2p_3/2_ and Ba3d_5/2_ spectra in the range of 770–810 eV, there is a partial overlap of the Co2p_3/2_ and Ba3d_5/2_ peaks around 778.4 eV, as well as a similar overlap between Co2p_1/2_ and Ba3d_3/2_ peaks around 793.7 eV (Fig. 2c, d). Deconvolution analysis allows for the extraction of individual peaks, with the Ba^2+^ peak exhibiting a slightly higher binding energy compared to the Co ions. The spin–orbit splitting of Co2p_3/2_ and Co2p_1/2_ was determined to be approximately 16.35 eV, indicative of Co being predominantly in the + 3 oxidation state. Additionally, the absence of satellite features around 787.1, and 803.2 eV rules out the presence of Co^2+^ on the surface^98,99^.

The O1s XPS peaks associated with the La_0.8_Ba_0.2_CoO_3_ material exhibit asymmetry (Fig. 2e). Upon employing deconvolution analysis, the observed asymmetrical peaks can be distinctly resolved into two components, each attributed to specific oxygen species. The lower-energy peak, situated at 528.87 eV and marked in green, is associated with O^2−^ ions, signifying the presence of lattice oxygen (O_L_). In contrast, the second peak, located around 529.55 eV and denoted by the purple line, corresponds to highly oxidative oxygen species, including O_2_^2−^ or O^−^ (O_vacancy_)^100,101^.

**Figure 3:**
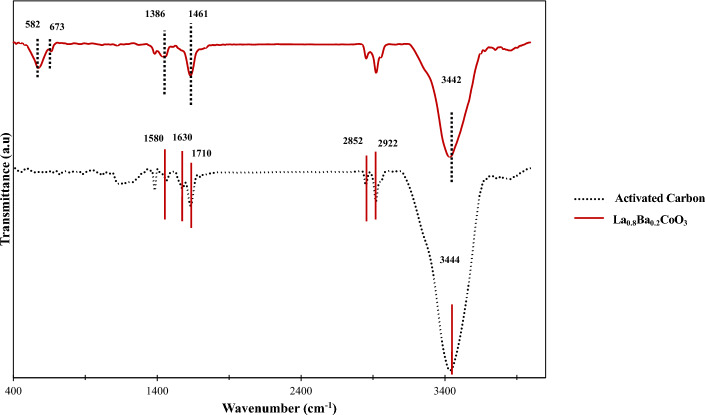
FTIR spectra Patterns of La_0.8_Ba_0.2_CoO_3_, and AC.

The FTIR spectra of La_0.8_Ba_0.2_CoO_3_ and AC (Fig. 3) provide information about the functional groups of the surface. For La_0.8_Ba_0.2_CoO_3_, the peaks observed in the 400 cm^−1^ to 700 cm^−1^ range can be attributed to the ABO_3_ perovskite structure. Additionally, a broad band at 3442 cm^−1^ is due to the physically adsorbed H_2_O molecules on the perovskite surface. In contrast, two low-intensity absorption bands at 1461 cm^−1^ and 1388 cm^−1^ corresponded to these water molecules’ O–H stretching, bending, and scissoring vibrational modes. The peak at 582 cm^-1^ is attributed to Co^3+^, and the peak at 673 cm^−1^ to Co^2+^, confirms the formation of the Co_3_O_4_ spinel structure. Following the XRD results, the impurities formed as BaCO_3_ are evident at 1388 cm^−1^ and 1461 cm^−1^, corresponding to the C–O bond's asymmetric stretching mode^102,103^.

The presence of oxygen functional groups on the surface is observed in the FTIR spectra of AC. The bands at 2800–3000 cm^−1^ are displayed in Fig. 3 (black line), demonstrating the presence of an aliphatic-CH stretching vibration (the peaks at 2922 cm^−1^ and 2852 cm^−1^ show both methylene (–CH_2_–) bridges and aromatic C–H stretching vibrations). The bands at 1710 cm^−1^ and 1580 cm^−1^ indicate the presence of carbonyl/carboxyl groups and an aromatic C=C ring stretching, respectively. The broad peak at 3444 cm^−1^ is attributed to the stretching O–H vibration (hydroxyl group), which is present due to the water utilization during the preparation process. Finally, the band at 1630 cm^−1^ is associated with hybridized C–C aromatic skeletal stretching of graphite^104,105^.

The SEM micrographs of La_0.8_Ba_0.2_CoO_3_ perovskite and AC illustrate crucial insights into the microstructure of perovskite and AC, shedding light on their respective surface properties and the potential impact on their electrocatalytic performance. (a-1, 2) presents the SEM images of La_0.8_Ba_0.2_CoO_3_, revealing a distinctive porous microstructure. The porous nature of La_0.8_Ba_0.2_CoO_3_ is a fundamental feature that significantly influences its overall performance as an electrocatalyst for the OER. This porous architecture results in an impressive specific surface area of 14 m^2^/g to put this into context, this specific surface area outpaces that of previously reported perovskite oxides commonly used for OER including Ba_0.5_Sr_0.5_Co_0.8_Fe_0.2_O_3_^106^, LaCo_1−x_Ni_x_O_3_^107^, LaFeO_3_^108^, Ca_x_La_1−x_Al_1−x_Mn_x_O_3_^109^, SrCo_0.95_P_0.05_O_3_^110^, BaZr_x_Fe_1−x_O_3_^111^. The extensive surface area of La_0.8_Ba_0.2_CoO_3_ provides a larger platform for electrochemical reactions and interactions with electrolytes, enhancing its electrocatalytic performance.

Furthermore, the SEM results indicate that La_0.8_Ba_0.2_CoO_3_ exhibits, irregular shapes, and rough surfaces, which are distinctive characteristics of its microstructure. This morphology is pivotal in facilitating electrochemical processes, as the increased surface roughness allows for more active sites for OER, promoting better catalytic performance.

In contrast, (b-1, 2) portrays the surface morphology of AC. AC displays a distinct surface texture characterized by uneven cavities and a clear porous structure. This unique microstructure imparts a high specific surface area to AC, a well-known attribute of AC materials. The high surface area is attributed to the presence of micro and mesopores, making AC an excellent candidate for electrochemical applications. The significant surface area of AC enhances its electron conductivity and electrochemical activity. This is particularly important in electrocatalytic applications, as the high surface area allows for a greater number of active sites where electrochemical reactions can occur. Furthermore, the porous structure of AC promotes effective mass transport of reactants and products to and from the electrode surface, which is crucial for efficient electrocatalysis^83^.

**Figure 4:**
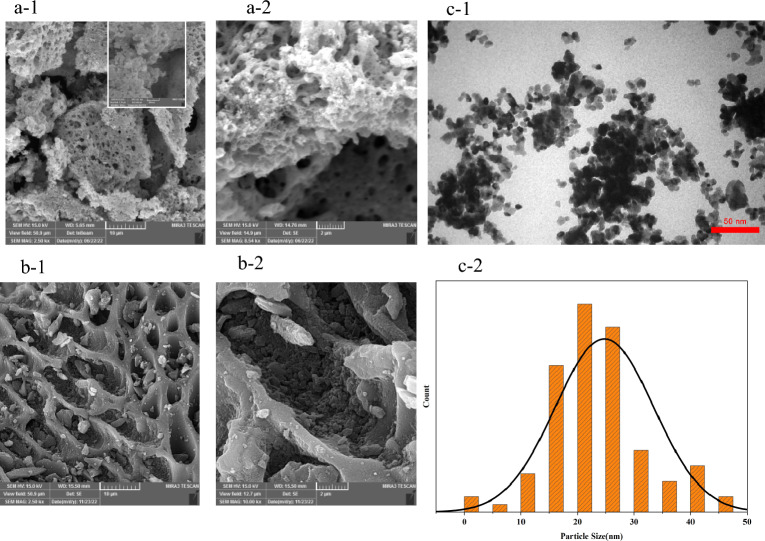
SEM images of the La_0.8_Ba_0.2_CoO_3_ (**a-1**, **a-2**), and AC (**b-1**, **b-2**). TEM analysis of La_0.8_Ba_0.2_CoO_3_ powder; (**c-1**): La_0.8_Ba_0.2_CoO_3_ nanoparticles, (**c-2**): particle size distribution.

In Fig. 4c-1,2, the TEM image of La_0.8_Ba_0.2_CoO_3_ nanoparticles reveals an irregular morphology characterized by distinct edges and facets. This irregular shape aligns with our observations in the SEM image. The observed roughness may be attributed to surface-related issues, such as defects, grain boundaries, or changes in chemical composition. Figure 4c-1 depicts the particle size distribution of La_0.8_Ba_0.2_CoO_3_ obtained through the analysis of various TEM images. The particle size has been estimated to be within the range of 1–50 nm, with approximately 54% of the particles falling within the 15–25 nm range. The average particle size is estimated to be around 25 nm^112,113^.

**Figure 5:**
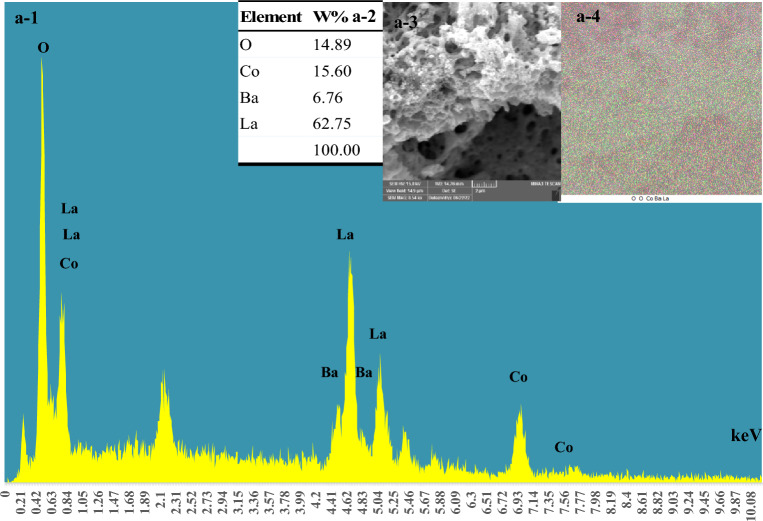
EDS spectroscopy and elemental mapping of La_0.8_Ba_0.2_CoO_3_.

Figure 5 represents the EDS spectroscopy and elemental mapping analysis of La_0.8_Ba_0.2_CoO_3_ (a-1,2,3, and 4). Table a-2 (insect in Fig. 5), displays the relative atomic concentrations of La, Ba, Co, and O through semi-quantitative EDS results, which are calculated using software based on an averaged signal from thousands of particles. The obtained catalyst compositions align closely with the calculated stoichiometric amounts of material used in the preparation process.

**Figure 6:**
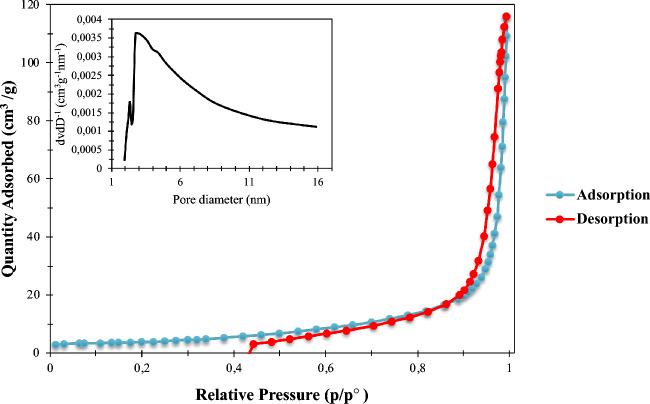
Isothermal N_2_ adsorption–desorption plot of La_0.8_Ba_0.2_CoO_3_.

The isotherm, pore size distribution curves, and the summarized BET report of the La_0.8_Ba_0.2_CoO_3_ are shown in Fig. 6 and Table 1, respectively. According to the IPUAC classification scheme, the obtained isotherm exhibits a type IV shape, accompanied by an H3-type hysteresis loop^114^. This type of hysteresis loop is typically observed in adsorption on mesoporous structures^115^. Furthermore, the pore size distribution curve displayed in the inset of Fig. 6 offers a more detailed view of the size distribution of these pores. It reveals that the majority of pores fall within a diameter range of 2.5 to 12 nm. This observation aligns seamlessly with the mesoporous structure of La_0.8_Ba_0.2_CoO_3_, further confirming the presence of these intermediate-sized pores. The availability of mesopores is of particular significance in electrocatalysis, as they provide an ideal environment for accommodating active sites and supporting electrochemical reactions..

**Table 1:** The summery report of the BET analysis of the La_0.8_Ba_0.2_CoO_3_.

Parameter	Value
BET Surface Area	14 m^2^/g
Micropore Area	2.3 m^2^/g
External Surface Area	12 m^2^/g

### RSM results

Table 2, provides a summary of the variables that RSM has assumed at each level. − Alpha, − 1, 0, + 1, and + Alpha were the five levels at which the variables were specified. The utilization of RSM at these specific levels is fundamental to the experimental design and analysis, enabling a comprehensive exploration of the variables and their potential effects on the study.

**Table 2:** The levels of the independent variables.

Variables	Levels					
	Unit	− Alpha	− 1	0	1	+ Alpha
Perovskite/active carbon (A)	–	1	1.608	2.5	3.391	4
KOH concentration (B)	M	0.1	0.282	0.55	0.817	1
Mass of PVDF (C)	mg	0.25	0.402	0.625	0.847	1

**Figure 7:**
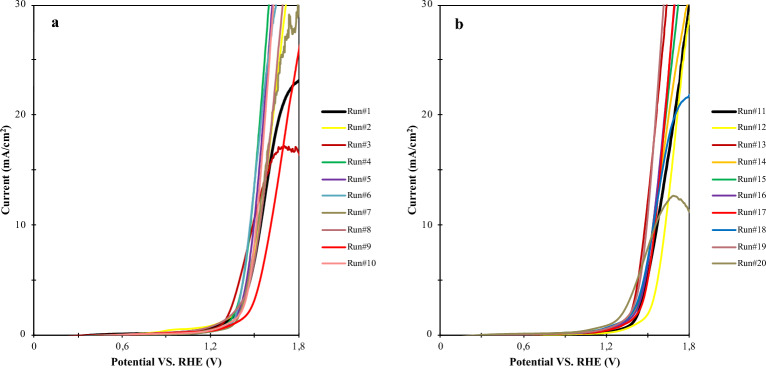
LSV curves of anodic material in OER based on CCD.

The acquired experimental conditions by RSM are listed in Table 3. Within this table, η_A_ represent the actual overpotential observed under experimental conditions, while η_P_ stands for the predicted overpotential derived from experimental design. Equation (1) was employed to determine the required overpotentials to achieve the current density of 10 mA/cm^2^ over composite electrode material from the polarization curves shown in Fig. 7. The figures, which are divided into two parts (a), showcasing runs 1–10 and (b), exhibiting runs 11–20, offer valuable insights into the electrocatalytic performance of the composite electrode material under various experimental conditions. The determination of the optimal overpotentials required to achieve the 10 mA/cm^2^ current density is enabled by this division.

**Table 3:** The determined experimental condition by RSM.

Std	15	3	11	6	10	2	20	1	5	13
Runs	1	2	3	4	5	6	7	8	9	10
Variables										
A	2.5	1.608	2.5	3.391	4	3.391	2.5	1.608	1.608	2.5
B	0.55	0.817	0.1	0.282	0.55	0.282	0.55	0.282	0.282	0.55
C	0.625	0.402	0.625	0.847	0.625	0.402	0.625	0.402	0.402	0.25
η_A_	315.45	307.45	262.09	301.61	269.79	243.14	294.2	310.4	385.95	284.67
η_P_	310.73	292.7	266.19	309.07	268.06	235.07	310.73	306.96	377.2	295.91

Std	7	14	12	17	16	19	8	18	4	9
Runs	11	12	13	14	15	16	17	18	19	20
Variables										
A	1.608	2.5	2.5	2.5	2.5	2.5	3.391	2.5	3.391	1
B	0.817	0.55	1	0.55	0.55	0.55	0.817	0.55	0.817	0.55
C	0.847	1	0.625	0.625	0.625	0.625	0.847	0.625	0.402	0.625
η_A_	341.68	400.9	258.32	317	310.33	314.76	325.21	314.44	266.38	334.2
η_P_	342.46	399.98	264.53	310.73	310.73	310.73	321.36	310.73	267.84	346.25

The proposed model for the response (overpotential) was developed using variance analysis (ANOVA) after evaluating the data obtained from experimental design and actual overpotentials. Subsequently, a quadratic model was selected as the most suitable method to predict the observed variations in the overpotential. The final equation, expressed in terms of coded factors, establishes the relationship between the independent variables and the response function^116^ (Eq. (4)).

4 $${\upeta }_{{\text{P}}}=310.73-23.25{\text{A}}-0.4917{\text{B}}+30.94{\text{C}}+11.76{\text{AB}}+0.9395{\text{AC}}-5.12{\text{BC}}-1.27{A}^{2}-16.04{B}^{2}+13.16{C}^{2}$$

Table 4 represents the ANOVA statistics for the overpotential model, which includes the correlation coefficient (R^2^), adjusted coefficient of determination, prediction coefficient, F-value, P-value, and %CV.

**Table 4:** The ANOVA results of developed model for overpotential of OER. *Source* Quadratic, R^2^ = 0.9618, Adjusted R^2^ = 0.9274, Predicted R^2^ = 0.9138, Adeq Precision = 49.73, C.V. = 3.47%

Source of Variations	SS	Df	MS	F-value	*p*-value	
Model	28,669.5	9	3185.5	27.95	< 0.0001	significant
Residual	1139.69	10	113.97			
Lack of fit	775.43	5	155.09	2.13	0.2133	not significant
Pure error	364.26	5	72.85			
Cor total	29,809.19	19				

SS, sum of squares; Df, degree of freedom; MS, mean square.

**Figure 8:**
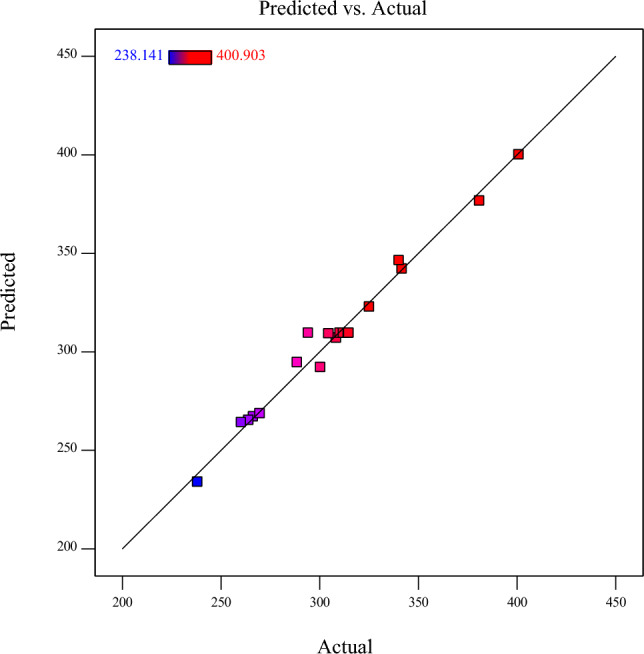
The residual normal probability for the OER overpotentials at 10 mA/cm^2^.

Based on the ANOVA results as presented in Table 4, the correlation coefficient of the model is 0.9618. This indicates that the suggested empirical model in Eq. (4) is capable of predicting over 96 percent of data, demonstrating a strong correlation between the independent variables and the response variable (overpotential). The adjusted coefficient calculated at 0.9274, further accentuates the significance and reliability of the suggested model. This adjusted coefficient takes into account the number of independent variables and the sample size, emphasizing the model's practical effectiveness in explaining the observed variations in the response variable. Furthermore, the model F-value, with a value of 27.95, attests to the model's statistical significance. This indicates that the model is indeed a meaningful and reliable tool for explaining the variation in the response variable. Importantly, when the associated P-values are below 0.0500, it signifies that the model terms are statistically significant in elucidating the variations in the response variable. Overall, the ANOVA results provide strong support for the assertion that the proposed model is endowed with exceptional predictive capabilities. The robust correlation coefficient, adjusted coefficient, and significant model F-value all validate the model's effectiveness in predicting the overpotential. Figure 8 illustrates the predicted values versus the experimental values for the OER overpotential response, further confirming the high validity of the RSM.

**Figure 9:**
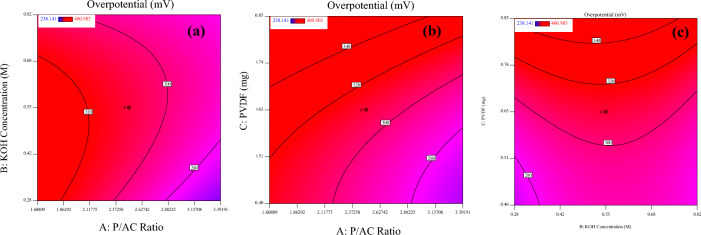
The effect of La_0.8_Ba_0.2_CoO_3_/AC ratio (**a**), PVDF amount in the composite material (**b**) and concentration of the KOH solution (**c**) on the needed overpotential for OER.

The effects of the three parameters on the response function can be visualized using response surface curves, as shown in Fig. 9. The figure shows that the increasing amount of PVDF in the composite material leads to an increase in the overpotential. This finding is consistent with the coefficient in Eq. (4) and can be attributed to the high solvent resistance and electrochemical stability of PVDF^82^. On the other hand, the overpotential is reduced by increasing the La_0.8_Ba_0.2_CoO_3_ /AC mass ratio. This parameter acts as a positive factor because it increases the active material in the composite. This finding aligns with the coefficient in Equation, indicating that a higher La_0.8_Ba_0.2_CoO_3_/AC ratio contributes to lower overpotentials. Additionally, the concentration of KOH positively affects the overpotential, meaning that increasing the concentration of KOH leads to lower overpotentials. This observation can be attributed to a high amount of OH- ions in the electrolyte, which enhances the electrocatalyst activity.

**Figure 10:**
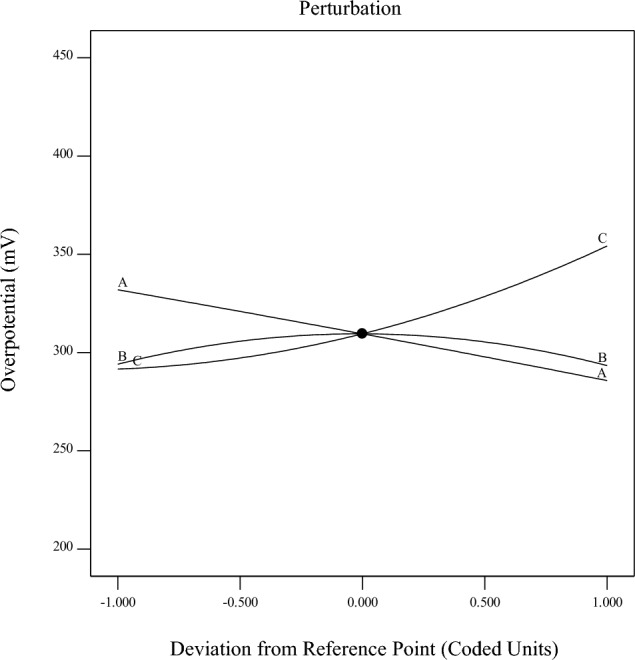
Perturbation plot for rate response (**A**: La_0.8_Ba_0.2_CoO_3_/AC ratio, **B**: PVDF amount and C: KOH concentration).

The perturbation plot, depicted in Fig. 10, served as a valuable tool for comparing the individual effects of the factors on the required overpotential at a specific point within the design space. This plot explains how changes in each factor influenced the overpotential response while keeping the other two factors constant. Upon close examination of the perturbation plot, it becomes evident that factors A and C have a positive effect on the overpotential. As these factors increase, the overpotential also increases, indicating a direct correlation. Conversely, factor B exhibits a negative effect on the overpotential. When factor B increases, the overpotential decreases, demonstrating an inverse relationship. Additionally, the relatively flat line for factor B in the perturbation plot suggests that its effect on the OER overpotential is less pronounced within the design space. This observation aligns well with the findings outlined in Eq. (4) and emphasizes that factor B plays a relatively minor role in influencing the overpotential in the specified experimental conditions.

### Electrocatalytic results of optimized electrode material

The three variables listed in Table 5 were determined through an optimization process to minimize the OER required overpotential over the electrocatalyst. The composite material was prepared with optimized La_0.8_Ba_0.2_CoO_3_/AC, PVDF amount, and KOH concentration values. Based on the optimization, the predicted overpotential for the optimal condition was found to be 308.22 mV. The actual overpotential was measured to be 301.20 mV upon the experimental testing, corresponding to a 2.27% error between the experimental and predicted values. The close agreement between the overpotential values indicates that the second-order model (Eq. 4) used to optimize the variables to achieve the lowest overpotential was appropriate and reliable.

**Table 5:** The actual and predicted overpotential at the optimum values of composite material.

	Perovskite/AC Ratio	CKOH (M)	Mass of PVDF (mg)	η_P_ (mV)	η_A_ (mV)	%Error
Composite materials	2.81	0.609	0.665	308.22	301.20	2.27

**Figure 11:**
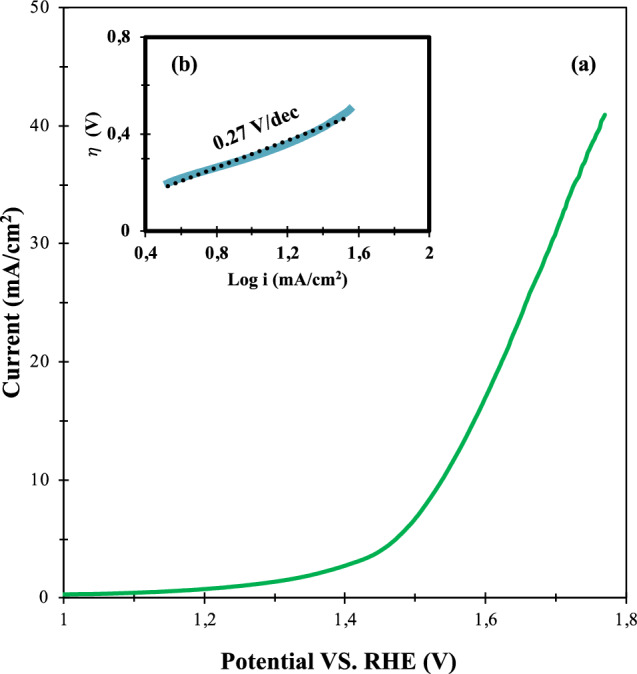
LSV (**a**) and Tafel (**b**) Curve at the optimized condition.

The electrochemical activity of optimized electrode material, La_0.8_Ba_0.2_CoO_3_/AC ratio (2.81), and PVDF Mass (0.665 mg), was evaluated by LSV measurements. These measurements were performed under the carefully chosen optimal conditions, including a KOH concentration of 0.609 M and a constant scan rate of 10 mV/s, as visually depicted in Fig. 11. Furthermore, the inset of Fig. 11 showcases the corresponding Tafel plot, derived from the LSV polarization curves.

In order to ensure the accuracy and validity of the results, the potential values obtained from the LSV tests were subjected to IR-correction and referenced to RHE. This referencing procedure is in line with established standards and conventions in the field, providing a common frame of reference for electrochemical measurements and facilitating meaningful comparisons across diverse studies.

A consistent pattern emerges when the data from the LSV curves obtained under these optimized conditions are compared with the performance of analogous anodic materials, as outlined in Table 6. A significantly lower overpotential and a markedly higher limiting current density are consistently achieved under the optimized conditions. These consistent observations collectively emphasize the clear enhancement of OER catalytic activity through the optimization of experimental parameters. This advancement represents a substantial step forward in the understanding and practical utilization of La_0.8_Ba_0.2_CoO_3_ electrocatalysis for the OER.

**Table 6:** Summary of the obtained overpotentials of perovskite- carbon composite electrocatalyst @ 10 mA/cm^2^.

Perovskite	Perovskite/ Carbon Material	Electrolyte	Limiting current (mA)	overpotential (mV)	References
La_0.8_Ba_0.2_CoO_3_	5.6:2	0.665 M KOH	45	301	This work
LaMnO_3_	4:1	0.1 M KOH	10–20	324	^117^
LaCoO_3_	5:1	1 M KOH	20	424	^35^
La_1−x_Ca_x_FeO_3_	3:2	0.5 M NaOH	4–12	~ 350	^118^
LaNi_1-y_Mg_y_O_3_	1:4.25	0.1 M KOH	15–28	450	^119^
LaNi_0.8_Fe_0.2_O_3_	1:4.25	0.1 M KOH	30–65	312	^120^
Ba_0.5_Sr_0.5_Co_0.8_Fe_0.2_O_3_	1:1	0.1 M KOH	28	~ 480	^106^
BaZr_x_Fe_1−x_O_3_	20:5	0.1 M KOH	20	412	^111^
(Ln_0.5_Ba_0.5_)CoO_3_ (Ln: Pr, Sm, Gd and Ho)	5:1	0.1 M KOH	10–12	~ 400	^69^
LaCo_0.2_Fe_0.8_O_3_	1:1	1 M KOH	25–75	340	^71^

Significant values are in bold.

**Figure 12:**
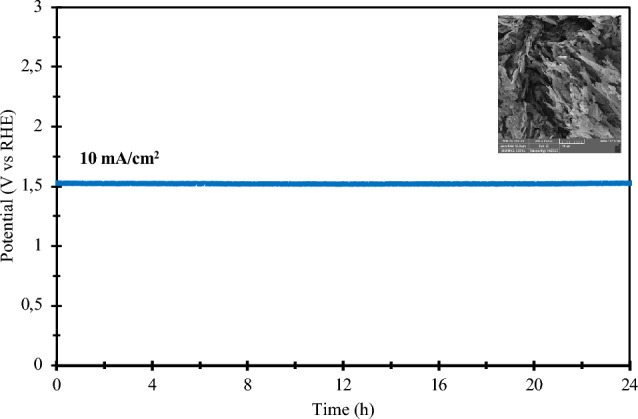
Stability result of the anodic material in 0.602 M KOH solution.

The study utilized chronopotentiometry testing to investigate the stability and reusability of the optimized electrode. In addition to its high electrocatalytic activity, the composite electrocatalyst exhibits commendable electrocatalytic stability for the OER. As illustrated in Fig. 12, the electrocatalyst maintains its electrocatalytic activity when subjected to a current density of 10 mA/cm^2^ for a continuous 24 h duration. Even after extended electrocatalysis, its morphology and microstructure remain unaltered, as confirmed SEM analysis (depicted in the inset of Fig. 12). Furthermore, a subsequent SEM analysis of the electrode's surface, conducted both before and after the 24-h test, unveils no discernible changes in the material. This substantial stability and resistance offer compelling evidence of the electrode material’s suitability for reuse under identical conditions. This stability can be attributed to the presence of catalytically active sites, facilitated by the inclusion of cobalt (Co), as well as the existence of oxygen vacancies. These factors collectively ensure the catalyst's resilience during the OER, thereby enhancing the material's overall stability.

## Conclusion

In conclusion, the experimental design approach using Design Expert 12.0.3.0 software successfully identified the optimal composition for preparing the electrode material and electrolyte solution to minimize the overpotential in OER. The CCD method was used to investigate the three effective parameters in the catalyst performance (the ratio of perovskite (La_0.8_Ba_0.2_CoO_3_) to carbon material (activated carbon), electrolyte concentration (KOH), and PVDF amount). The perovskite was prepared using the sol–gel method, and various characterization techniques, including XRD, SEM, FT-IR, and N_2_ adsorption–desorption analyses, were performed to analyze the structure and properties of the perovskite material. The low and high ranges of the parameters were determined based on previous experience. Design Expert software generated a set of 20 experiments for the three variables. The response variable chosen was the required overpotential to achieve a current density of 10 mA/cm^2^. The Quartic second-order model established the relationship between independent and response variables. The ANOVA results indicated that the model was significant, with a high correlation coefficient, adjusted coefficient, F-value, and low *P*-value.

Furthermore, the optimized material has a 301.2 mV overpotential, slightly lower than the predicted overpotential, with a small error of 2.27%. Additionally, the stability of the composition electrode material suggests that it is a good candidate anodic material for OER. Overall, the combination of experimental design, statistical analysis, and characterization techniques allowed for identifying an optimized electrode material composition with improved OER performance and stability, providing a promising candidate for electrocatalytic applications. As a prospective direction for future research, an investigation into the activity of the La_0.8_Ba_0.2_CoO_3_ perovskite for the CER, utilizing an online Gas Chromatography (GC) device, is recommended. This avenue shows great promise in advancing the field of electrocatalysis for water splitting. The significance of this forthcoming research effort lies in its ability to address a critical challenge in water splitting processes—the non-selective nature of commonly used electrocatalysts for both the CER and the OER. Achieving selectivity is paramount for enhancing the efficiency and economic viability of water electrolysis, a pivotal component of clean hydrogen production.

## Data Availability

All data generated or analysed during this study are included in this published article.
